# Estimating Biomass and Vitality of Microalgae for Monitoring Cultures: A Roadmap for Reliable Measurements

**DOI:** 10.3390/cells11152455

**Published:** 2022-08-08

**Authors:** Michael Schagerl, Rainer Siedler, Eliška Konopáčová, Sameh Samir Ali

**Affiliations:** 1 Department of Functional and Evolutionary Ecology, University of Vienna, Djerassiplatz 1, A-1030 Vienna, Austria; 2 Institute of Environmental and Chemical Engineering, University of Pardubice, Studentská 573, 530 12 Pardubice, Czech Republic; 3 Institute of Hydrobiology, Biology Centre v.v.i., Czech Academy of Sciences, Na Sádkách 702/7, 370 05 České Budějovice, Czech Republic; 4 Biofuels Institute, School of the Environment and Safety Engineering, Jiangsu University, Zhenjiang 212013, China; 5 Botany Department, Faculty of Science, Tanta University, Tanta 31527, Egypt

**Keywords:** algae, growth, biotechnology, cultivation, biomass, fluorescence

## Abstract

Estimating algal biomass is a prerequisite for monitoring growth of microalgae. Especially for large-scale production sites, the measurements must be robust, reliable, fast and easy to obtain. We compare the relevant parameters, discuss potential hurdles and provide recommendations to tackle these issues. The focus is on optical density and in vivo autofluorescence of chlorophyll, which have proven to be ideal candidates for monitoring purposes. Beyond biomass, cell vitality is also crucial for maintaining cultures. While maximizing biomass yield is often the primary consideration, some applications require adverse growth conditions for the synthesis of high-quality compounds. The non-invasive technique of pulse-amplified modulated (PAM) fluorescence measurements provides an ideal tool and is increasingly being employed due to ever more affordable devices. We compared three devices and studied the robustness of the dark fluorescence yield of photosystem II (Fv/Fm) at various cell densities. Although the so-called inner filter effects influence the fluorescence signal, the resulting Fv/Fm remain stable and robust over a wide range of cell densities due to mutual effects.

## 1. Introduction

The determination of microalgal biomass is an essential component of cell cultivation and is commonly required in biotechnology, medicine and other disciplines [1]. Assessing the status of a culture and its growth performance calls for the rapid and efficient estimation of biomass. Various options are available to estimate biomass [2], with the selection depending on the focus of interest. For applied research and commercial cultivation, the biomass parameters must be robust, reliable and easy to measure. Importantly, the result must be quickly available as a fast decision-making tool, e.g., for starting the harvest or modifying the current culture conditions. Given a population doubling time of less than one day [3,4], the availability of such data on the following day may already be too late for operations.

The parameters for obtaining biomass estimates of microalgae can be categorized according to their relation to biomass and based on their units (Table 1). It is impossible to measure fresh mass of aquatic microalgae directly. In unialgal cultivation systems, algal dry mass (DM) is the primary biomass parameter, with dense photoautotrophic cultures comprising around 10 g L^−1^ DM. DM is given in absolute units based on area or volume. Another group of common methods is also based on absolute units, but they consider only part of the algal biomass (carbon content, in vitro chlorophyll-a = chl-a); this results in variations depending on the strain used and on the culture conditions provided. Other indirect absolute parameters are the cell number and the algal biovolume. Although algal biovolume does not consider specific gravity, it additionally takes cell size into account, which increases accuracy. Estimations resulting in absolute values provide several advantages. They are based on volumetric or areal units, which enable comparisons between species, between treatments and different cultivation systems. The most common proxies for routine measurements are, however, a third group of parameters providing relative values. Measuring optical density (OD) and in vivo autofluorescence of chl-a (IVF) are standard procedures for monitoring purposes. These derived, relative parameters are not suited for a direct comparison between species and different treatments. They should be applied with caution and only within single cultivation approaches. 

**Figure 1:**
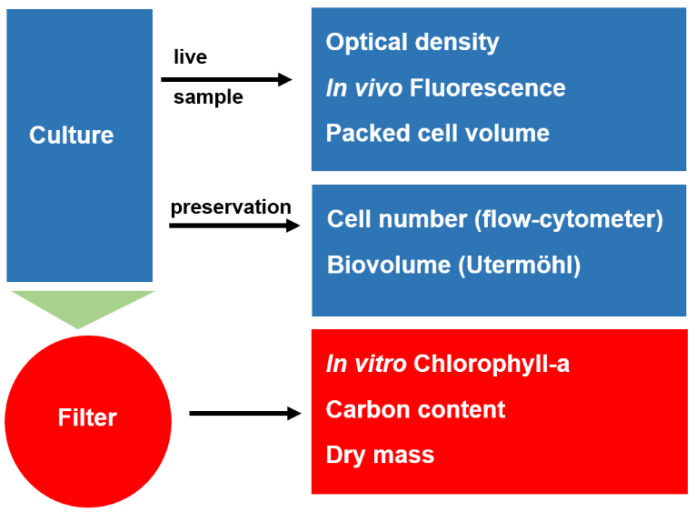
Sample treatment for estimation of microalgal biomass.

Depending on the parameter used, specific sample pretreatment may be required. Easy-to-use parameters such as OD and IVF require only a few mL of raw sample, whereas other methods require sample preservation and some cases also concentration (Figure 1). Such additional steps always carry the risk of misapplication.

Beyond knowing the algal biomass, information on cell vitality is crucial for cultivation. Maximizing the biomass yield goes hand in hand with providing optimal growth conditions, but some applications call for suboptimal conditions. One prime example is two-stage cultivation, which includes an additional step providing adverse growth conditions to enhance the synthesis of high-value compounds [5,6]. One method to gain insight into the vitality of photoautotrophs is pulse-amplified modulated (PAM) fluorescence of photosystem II. PAM-fluorescence was initially developed for field crops [7,8] and later adapted to other photoautotrophs [9]. It is a fast and non-invasive technique to measure the overall performance of photochemical processes in plants [10,11], and is increasingly used for commercial microalgae cultivation [12,13,14,15].

The current study provides an overview of routine parameters for estimating microalgal biomass. We conducted experiments to highlight advantages and disadvantages of the respective parameter. Choosing the appropriate method for microalgal biomass determination always depends on the question of interest. No method fits all requirements: each approach has advantages and disadvantages [16]. Whereas benefits of methods are always highlighted in manuals and reports, challenges and negative results are usually ignored thus complicating their correct application. This so-called publication bias is, however, a general problem in science and not restricted to technical papers [17,18,19]. To clearly show potential pitfalls of algal biomass estimation, we included examples of incorrect measurements.

Estimating microalgal biomass remains a challenge because the cells are usually dispersed in the medium. Direct gravimetrical measurements—which require concentration of cells—are therefore not possible. Indirect estimations based on turbidity (OD) or fluorescence (IVF) bear the risk of producing artefact-caused interferences between cells and light. We put special emphasis on OD and IVF here because they are commonly used for monitoring purposes in commercial cultivation systems. Based on our own experiments, we discuss pitfalls, which are mainly caused by high cell density, and we provide recommendations to overcome these problems. We have also included the PAM-fluorescence technique here because this method is increasingly being applied in large-scale systems. We specifically focus on the question whether this method is susceptible to high cell concentrations, which often cause serious problems in other photometric techniques.

## 2. Materials and Methods

We designed various experiments for evaluating methods of algal biomass estimation; these are explained in the subsequent section after the description of the methods.

### 2.1. Methods Applied

#### 2.1.1. Dry Mass

A defined volume of the algal suspension is filtered with gentle vacuum-filtration on pre-combusted, pre-weighed glass fiber filters (Whatman GF/C). Filters are then dried for 24 h in a drying cabinet at 95 °C. Thereafter, the filters are cooled down in a desiccator, re-weighed and the DM is calculated. DM (mg L^−1^) = (filter with dried material (mg) − pre-combusted filter (mg)) × filtered sample volume^−1^ (L).

#### 2.1.2. Packed Cell Volume (PCV)

The method was adapted from hematocrit measurements [20,21]. We used hematocrit tubes to determine the PCV of algae (TPP tubes, Buch & Holm, Herlev, Denmark). A defined volume of algae suspension is pipetted in TPP tubes and then centrifuged. Cells heavier than the surrounding medium congregate in the capillary of the tube, which allows volumetric measurement of the cell pellet with a specific TPP “easy read” device.

#### 2.1.3. Algal Cell Number and Biovolume

Immediately after harvest, 50 mL of algae suspension are preserved with 2–3 drops of Lugol’s Iodine solution until the sample is the color of beer (preparation: 10 g KI are dissolved in 20 mL distilled water, 5 g iodine is added and dissolved, then 50 mL of 10% acetic acid is added). A defined volume is transferred to counting chambers and analysed by an microscope [22,23]. Depending on the cell density, Utermöhl sedimentation chambers and inverted microscopes can be taken (low cell density) or Neubauer chambers or similar types can be used for compound microscopes (high cell density). First, counting units are classified according to their morphology (cells, colonies, filaments) and size. Then each unit is counted separately. Depending on the shape of counting units, geometric formulas are used to calculate a mean biovolume of the respective size class [24,25]. Multiplying the cell number with the mean biovolume of the size class and adding the biovolumes of the categories, yields the overall algal biovolume. Assuming a specific gravity of one, the biovolume can be converted into biomass.

#### 2.1.4. Carbon Content

A defined volume of the suspension is filtered onto pre-combusted and pre-weighed glass fiber filters (Whatman GF/C, Hillsboro, OR, USA). To remove all inorganic carbon, filters are rinsed with 5% HCl to remove precipitation, followed by rinsing with MilliQ-water. After drying at 60 °C, filters are packed in aluminum foil and analyzed in an elemental analyzer (Vario MICRO Cube, Hanau, Germany).

#### 2.1.5. In Vitro Chlorophyll-a

After filtration of a defined volume of the culture (Whatman GF/C), the filters are packed in aluminum foil and frozen at −20 °C to assist cell burst. Filters are then homogenized in 90% acetone with an ultrasonicator (Branson Sonifier 250, Danburg, CT, USA) and left for extraction of photosynthetic pigments overnight in the refrigerator. The extract is centrifuged, decanted and the supernatant is measured in the spectrophotometer against acetone at 663 nm. For calculation, the published extinction coefficient for 90% acetone was used [26]: Chl-a [µg L^−1^] = 11.41 × absorbance 663 nm × v_ext_ × V_sample_^−1^ × d^−1^. v_ext_ = volume of the extract (mL), V_sample_ = sample volume (L), d = cuvette path length (cm).

#### 2.1.6. In Vivo Chlorophyll-a

IVF of algae suspensions is measured by means of spectrofluorometry (HORIBA Fluorolog-3, Kyoto, Japan) with an excitation wavelength of 410 nm and the emission wavelength set to 670 nm [27]. Pure medium is taken as a reference value.

#### 2.1.7. Optical Density

OD of algae suspensions is measured spectrophotometricically (Hitachi U-2001, Tokyo, Japan) at 750 nm with pure medium set as a reference.

#### 2.1.8. Pulse-Amplified Modulated Fluorescence

The maximum dark fluorescence yield of PSII (Fv/Fm) serves as a proxy of the overall photosynthetic efficiency [28]. Maximum values of non-stressed eukaryotic photoautotrophs are highly conserved and range between 0.7 and 0.8 [13]. For cyanobacteria, Fv/Fm is around 0.4 and 0.6 [29] because phycobiliproteids interfere with the fluorescence signal [30,31]. Life samples are placed in a cuvette and pre-darkened for 10 min. Then minimum fluorescence (F_0_) is obtained, followed by measurement of maximum inducible fluorescence during a light-saturating flash (F_m_). Fv/Fm is calculated with variable fluorescence (Fv) = F_m_ − F_0_. For instruments used, refer to Section 2.2.7. “Algal Cell Vitality”.

### 2.2. Experiments Conducted

#### 2.2.1. Carbon Content

The experiment was conducted to assess the robustness of cellular carbon as a proxy for algal biomass at different irradiances and nitrogen supply. *Anabaena torulosa* was grown in bubble column reactors supplied with sterile air at 25 °C. Four different settings were provided; three replicates were cultivated for each treatment: BG11 full medium [32] combined with high-light (75 µmol photons m^−2^ s^−1^) and low irradiance (30 µmol photons m^−2^ s^−1^), BG11 medium without nitrogen supply under high- and low-light conditions. A light–dark cycle was set to 12 h:12 h. Cultures were harvested after 4 and 9 days.

#### 2.2.2. In Vitro Chlorophyll-a

Robustness of in vitro chl-a was tested with growth experiments of *Limnospira fusiformis* cultivated in full Zarrouk medium [33] and under nitrate-limited conditions, respectively (the equivalent amount of NaCl was added instead NaNO_3_). To avoid nitrogen carry-over, pre-cultures with reduced NaNO_3_ (20% of the initial concentration) were run for a few days for acclimation. Just before the main experiment started, filaments were rinsed with the respective medium and resuspended. We cultivated in bubble column reactors and harvested after 4, 8 and 11 days (4 replicates for each treatment; 27 °C, 50 µmol photons m^−2^ s^−1^; LED panels, light:dark cycle = 12 h:12 h).

#### 2.2.3. Packed Cell Volume and Algal Biovolume

PCV and algal biovolume against DM were measured to test their applicability as monitoring parameters in a growth experiment of *Limnospira fusiformis*. We used bubble column reactors supplied with sterile air (n = 9). Cultivation condition was Zarrouk medium at 25 °C, 40 µmol photons m^−2^ s with LED panels, light:dark cycle = 12 h:12 h). Measurements were done every two days.

#### 2.2.4. Optical Density

Two experiments were conducted: (1) a comparison between microalgae of different morphology and (2) an experiment with and without sample dilution.

(1) Unialgal batch cultures of *Chlorella vulgaris* were grown in 2 L cultivation flasks placed on magnet stirrers. Growth conditions were BG11 medium at 22 °C and permanent light of approximately 150 µmol photons m^−2^ s^−1^ reaching the bottle’s light-facing side (fluorescent tubes, Lumilux Cool White, 36 W/840, Osram, Munich, Germany). Sterile air enriched with 5% CO_2_ was delivered to the bottom of the flask via a silicone hose. *Limnospira fusiformis* was cultivated in Zarrouk medium in bubble column reactors run with sterile air at 27 °C and 50 µmol photons m^−2^ s^−1^ and a light:dark cycle = 12 h:12 h (warm-white LED panels).
(2) *Anabaena catenula* was grown at 25 °C in bubble column reactors aerated with sterile air. Continuous light supply was provided via warm-white LED panels and 30 µmol photons m^−2^ s^−1^. Culture treatments consisted of four groups with different nitrogen supply (3 replicates each). The basic growth medium was BG11 but without inorganic nitrogen (treatment 1 with atmospheric N). Three more N sources were added to the basic growth medium for the respective treatment as “pulses” every 24 h: 1 mM NaNO_3_, 1 mM NH_4_Cl, 0.5 mM CH_4_N_2_O.

#### 2.2.5. In Vivo Chlorophyll-a

*Chlorella vulgaris* and *Haematococcus lacustris* were grown in bubble column reactors in BG11 medium supplied with sterile air, continuous irradiance supply with 30 photons m^−2^ s^−1^ at 25 °C. After 10 days, cells were further condensed by centrifugation at 750 RPM for 10 min to increase cell density. The highly condensed samples were then diluted with sterile medium and repeatedly measured for the dilution series.

#### 2.2.6. Comparison of Biomass Parameters

*Limnospira fusiformis* was cultivated in bubble column reactors supplied with sterile air (n = 9). Cultivation conditions were Zarrouk medium at 25 °C, 40 µmol photons m^−2^ s^−1^ with LED panels, light:dark cycle = 12 h:12 h). Measurements were done on days 0, 3, 5 and 7. Doubling times (td) and growth rates (µ) were calculated with the respective biomass parameters as follows: µ = (ln X_t2_ − ln X_t1_)/(t_2_ − t_1_) and td = ln2/µ with X = biomass parameter and t = time of harvest (d).

#### 2.2.7. Algal Cell Vitality

The experiment was designed to study possible interferences of high algal biomass with fluorescence signals. In addition, we compared three different PAM-fluorescence devices to test compatibility of Fv/Fm with different types of equipment concerning its accuracy. *Chlorella vulgaris* was cultivated in bubble column reactors (sterile air) in BG11 full medium at 25 °C and continuous irradiance supply (40 µmol photons m^−2^ s^−1^, provided with warm white LED panels) to an OD of 13. From the dense culture, dilutions were conducted with BG11 medium.

PAM 2500-1 (PAM-2500/US-C, Walz company, Effeltrich, Germany) is equipped with an emitter unit (2500-E), a high-sensitivity detector (2500-D) and a cuvette holder (ED-101US/MD). PAM2500-2 (Walz company, Effeltrich, Germany) is a standard instrument for higher plants, equipped with fiber optics for measurements on leaves of higher plants and macroalgae. We used the add-on suspension cuvette KS-2500. The AquaPen AP100 (Photon Systems Instruments company, Drásov, Czech Republic) is a small battery-operated device and quite inexpensive compared to highly sophisticated laboratory instruments. PAM2500-1 is a device specifically designed for laboratory experiments of low-concentrated algae communities and mainly used in basic research; PAM2500-2 is a robust instrument for applied research and routine measurements and can be taken to the field. The AP100 device is a low-budget instrument suited for monitoring purposes.

## 3. Results and Discussion

### 3.1. Dry Mass

DM is the primary parameter to measure algae biomass in cultures. With a few exceptions (diatoms, coccolithophorids and synurophytes), DM comprises around 80–90% organic matter (ash-free dry mass) and 10–20% inorganic material (ash mass) [2,34]. Ash-free dry mass was determined to be an ideal proxy for the energy content of organisms [35], but it involves additional working steps and requires further time for analysis. DM of suspended microalgae is generally determined gravimetrically after centrifugation or filtration followed by drying [2,36]. Filtration is superior to centrifugation because the latter works only if the cells have a higher specific gravity than the surrounding medium. Moreover, decanting of the supernatant can potentially cause substantial loss of material. Filtration also involves certain hurdles: compared to the filter itself, the filtrated material has low weight. An improper filtration technique might therefore result in negative DM due to loss of filter material. Low cell density combined with low filtration volumes exacerbates this problem. Moreover, the small weight differences set a high standard for the accuracy of weighing equipment. Another pitfall is clogging of filters at high cell density. As low filtration volume increases the pipetting error, dilution of the suspensions before the filtration step is highly recommended. Summarizing, DM is not suited for daily monitoring because of the delayed availability of the results.

### 3.2. Carbon Content

**Figure 2:**
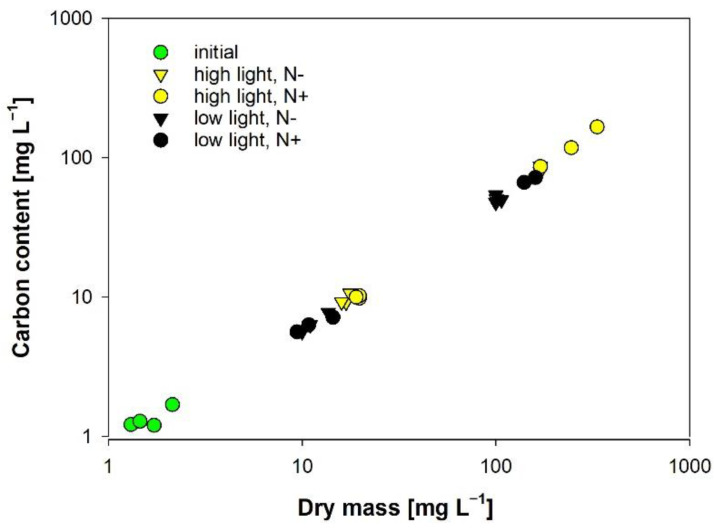
The carbon content of *Anabaena torulosa* grown in full BG11 medium (N+) and in nitrogen-depleted medium (N−) and under high and low light is about 50% of the total DM (r^2^ = 0.99, n = 27).

We found carbon content of algal biomass to be very stable irrespective of the treatment; cultures provided with inorganic nitrogen and high irradiance supply performed best (Figure 2). Even under nitrogen-limiting conditions, carbon content remained stable, which was is in agreement with other studies [37]. Although carbon content is highly related to algal biomass, carbon quantification for monitoring purposes is too laborious and expensive in terms of hardware costs. Additionally, filtration onto pre-combusted filters is required before analysis, calling for certain precautions (see previous paragraph).

Carbon contributes the most to DM and is mainly of high interest for ecological research issues such as energy fluxes in wood webs [38]. However, only a few studies are available providing carbon-to-biovolume conversions [39,40]. Importantly, carbon content is stable within algal classes, but between groups substantial differences may exist, especially for diatoms [41]. Finally, differences between freshwater and marine species were observed, with an inverse relationship of carbon-to-cellular biovolume in marine specimens [40].

### 3.3. In Vitro Chlorophyll-a

**Figure 3:**
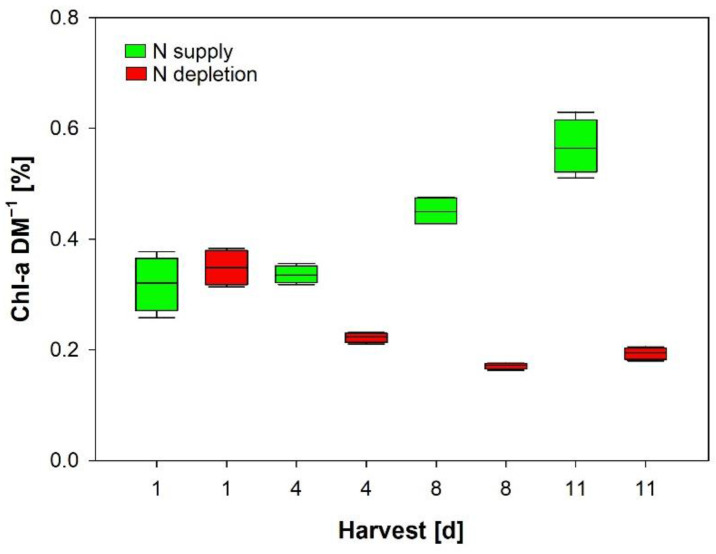
Significant changes of in vitro chl-a per DM (percent values) during cultivation of *Limnospira fusiformis* supplied with full Zarrouk medium and in nitrogen-depleted medium (rm-Anova with time and nitrogen supply as factors). Mean and standard deviation (n = 4).

Our experiment revealed large variations of in vitro chl-a per DM (Figure 3) depending on growth conditions, which is in agreement with other studies [37,42]. The experiment clearly revealed that nitrogen depletion results in reduced specific Chl-a concentration (nitrogen is an element of the Chl-a molecule). Nitrogen supply enables high growth rates, thus increasing turbidity due to high cell densities. Cells react in increasing the cellular pigment content for optimal light absorption (Figure 3).

Chl-a is highly influenced by the physiological state, age of cells and growth conditions provided [43]. In natural populations, chl-a per unit biovolume varies by about two orders of magnitude [44,45]. Additionally, choosing different analytical procedures has a major influence on the result [46,47,48]. In some manuals, spectrophotometric chl-a analysis without cell disruption is described [49], but this results in significantly lower contents especially for algae with rigid cell walls [46]. Although in vitro chl-a is taken as a proxy for biomonitoring and in assessing the trophic status of water bodies [50] in the absence of any simpler alternative, it is not recommended for daily routine measurements in algal production sites.

### 3.4. Packed Cell Volume and Algal Biovolume

**Figure 4:**
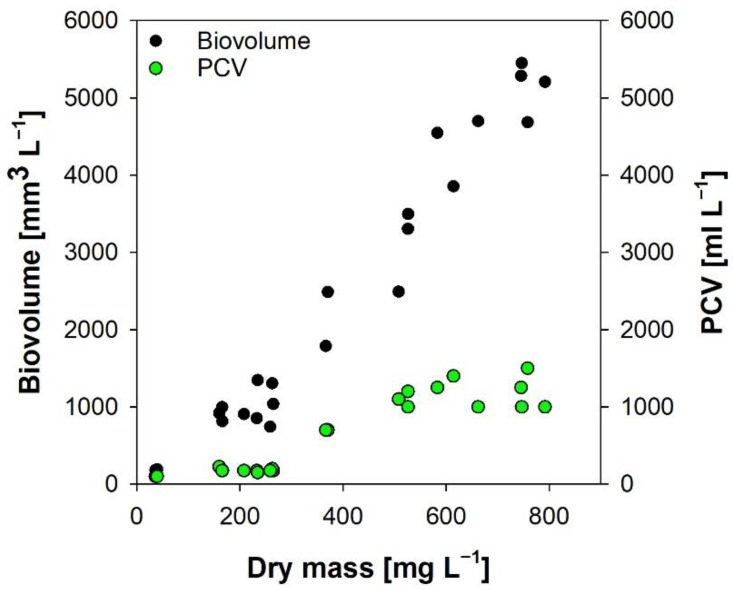
Relation of DM to biovolume and PCV. *Limnospira fusiformis* was cultivated for one week.

According to our experience (this experience and unpublished data), PCV can only be taken as a very rough estimate of algal biomass; the correlation between PCV and DM was moderate (Figure 4, r^2^ = 0.81, n = 25). Besides imprecise readings from the hematocrit “easy read” device, cell shape and density are critical factors. Simple and heavy forms will aggregate as compact pellets (*Chlorella*), whereas expanded, complex shapes (e.g., spirals of *Limnospira*) will result in low packing density; therefore, PCV cannot be converted into algal biovolume. Moreover, as centrifugation only works with cells heavier than the surrounding medium, specimens with low density will not settle due to buoyancy, thus resulting in low PCV. This presents an additional potential problem with organisms containing gas vesicles or lipid droplets. Exactly this phenomenon is observed in our experiment with *Limnospira*-containing gas vesicles (Figure 4). Notwithstanding, PCV in hematocrit tubes has already been applied for overall estimates of algal biomass [51,52,53].

Our growth experiment with *Limnospira fusiformis* comparing DM and biovolume revealed a strong relationship (Figure 4, r^2^ = 0.96, n = 25). Mixed cultures, large variations in cell size and complex cell shape will however reduce the relation with DM. Modifying growth conditions also results in less correlation [54,55]. Moreover, biovolume estimations are prone to failure because the results largely depend on the researcher’s work experience.

Algal biovolume is obtained by direct microscope counts either by means of an inverted microscope and settling chambers [23,25] (low cell density), or with hematocytometer counting devices (high cell density) [56]. Manual biovolume estimations take a vast amount of time. The simple geometric cell shapes of most cultures used for commercial exploitation and carefully following the recommendations [24,25] help minimize this challenge. The cell number is sometimes counted automatically by means of flow-cytometry with high precision [57]. Absolute biovolumes from these automated routines cannot easily be obtained, although successful efforts already have been undertaken with simple cell shapes by implementing data from the scatter pulse areas [58,59].

### 3.5. Optical Density

**Figure 5:**
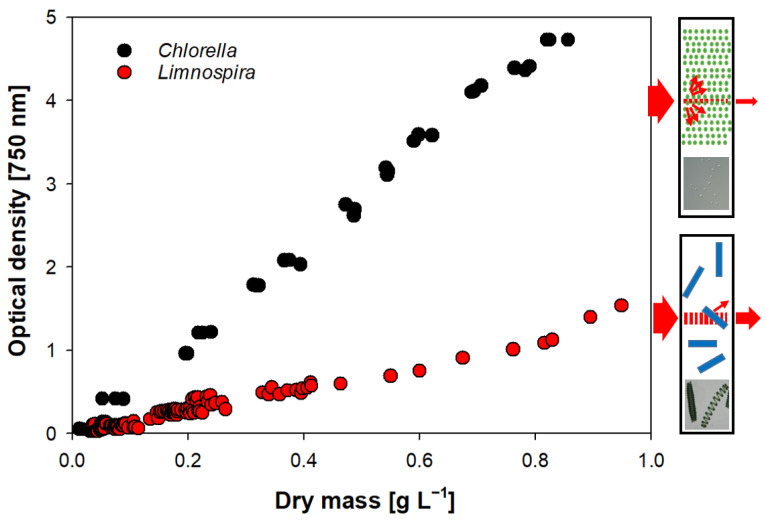
Comparing OD of two species *Chlorella* sp. and *Limnospira fusiformis.* Sketches on right: cuvette cross sections with higher OD of *Chlorella* caused by increased scattering due to many small cells (top) compared to the large filaments of *Limnospira* (bottom).

We found a high correlation between OD and DM within single cultures (Figure 5). As long as cultures share the same properties, extrapolation to DM can easily and precisely be performed after calibration. Between species, however, a direct OD comparison is not justified. Smaller cells show a higher OD compared to large forms due to higher scattering, as seen in our experiment (Figure 5).

**Figure 6:**
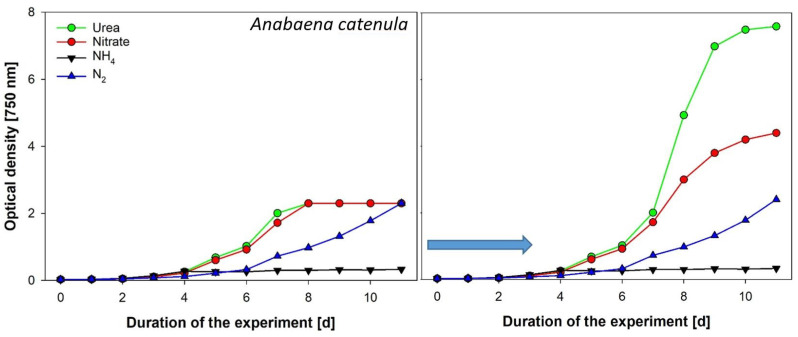
Growth experiment with the cyanobacterium *Anabaena catenula* supplied with different nitrogen sources (means of 3 replicates). **Left**: OD was measured without considering the upper absorbance limit of the spectrophotometer. Erroneous results are shown from which incorrect conclusions might be drawn. **Right**: samples were diluted at absorbance values > 1 (indicated by the blue arrow) and then multiplied with the dilution factor.

OD is based on measuring the loss of intensity of transmitted light due to the specific absorption and scattering properties of cells. Caution must be taken for dense cultures; otherwise, incorrect conclusion might be drawn from the instrument readings, if they reach the upper detection limit of devices, as seen in our example (Figure 6). Very high cell densities with OD > 1 are problematic for the reliability of the results because low transmissions pose a challenge to sensor sensitivity. As absorbance (A) units are based on a logarithmic scale, A = 1 is 10% of incident light transmitted, A = 2 is 1%, and A = 3 is 0.1%. The latter value is close to the upper absorbance range of many spectrophotometers on the market (Figure 6). The recommendation is therefore to dilute samples with A > 1 to increase the informative value of the measurements.

OD is widely used to monitor algae growth. It is a fast, reliable and inexpensive method independent for the experience of the worker. It can also be carried out by non-scientific staff. OD is a turbidity measurement based on light scattering of suspended particles. OD provides an overall value of particle density, size, turbidity, and absorption properties. This parameter also encompasses precipitates, dead organisms and microbial contaminants, which calls for cross-validation with other methods such as microscopy or IVF. For microalgae cultures, either 550 nm or 750 nm are used to exclude absorption interferences with photosynthetic pigments, because such pigments show large variations depending on the physiological stage of the organisms [60]. Strangely enough, OD measurements at the absorbance maximum of living algae at around 680 nm have also been proposed in the recent past [16,61].

### 3.6. In Vivo Autofluorescence of Chlorophyll-a

**Figure 7:**
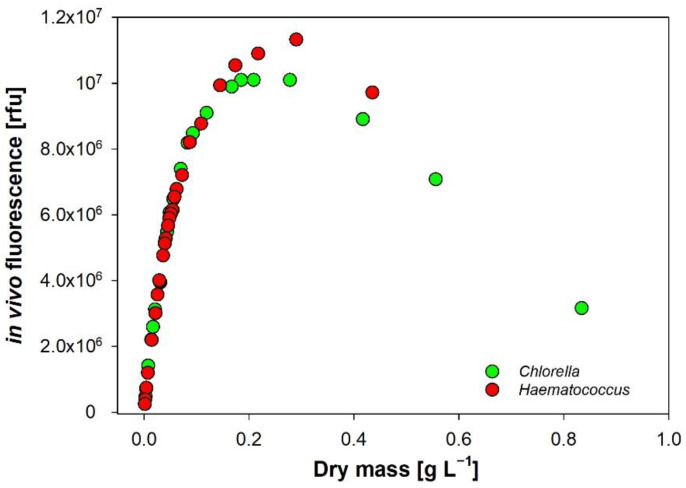
Dilution series of two commercially exploited chlorophytes *Haematococcus* and *Chlorella* (instrument HORIBA Fluorolog-3). The correlation at low to medium cell densities is linear, followed by a saturation and a drop of the fluorescence signal at high and maximum densities, respectively.

**Figure 8:**
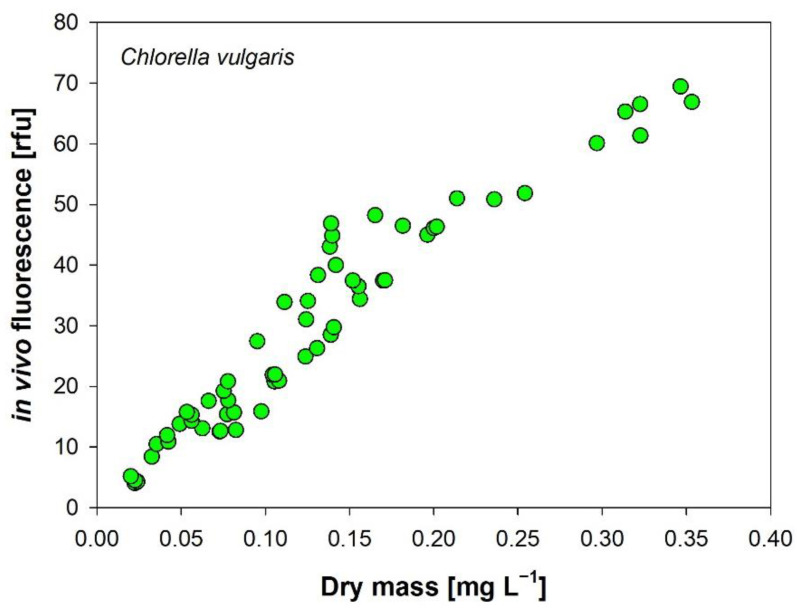
Growth of *Chlorella vulgaris* monitored by DM and in vivo chlorophyll autofluorescence (instrument SHIMADZU RF-5301PC). Samples with DM > 0.25 g L^−1^ (corresponds to OD ~ 0.5) were diluted with medium before measurements and the values multiplied by the dilution factor.

We found a strong linear correlation of the fluorescence signal at low to medium concentrations, followed by a dampening and saturation and an unexpected drop of the signal at the highest cell densities (Figure 7). This drop is explained by the so-called primary and secondary inner filter effect (IFE), which are well documented for dissolved substances, such as dyes and fluorophores [62,63,64]. Along the light path, the excitation beam is partly absorbed and scattered by cells, which lowers the excitation energy over the optical path length. Since the fluorescence signal is proportional to the excitation intensity, the resulting emission will be decreased (primary IFE). The emitted fluorescence signal is then further reduced by absorption and scattering of particles (secondary IFE). The recorded fluorescence underestimates algal biomass; the higher the OD, the larger this measuring artefact is. The unintended IFE can be minimized by timely dilution of the raw sample, as shown in our dilution experiment (Figure 8).

All oxygenic photoautotrophs contain the natural fluorochrome chl-a. The pigment is widely used as a proxy for biomass of in situ measurements of phytoplankton [65,66] and sometimes also for algal cultivation [16], although a blurriness similar to that with the spectrophotometric method is evident. Interestingly, IVF turned out to be a better predictor for algal biomass than in vitro chl-a, which was explained by a stronger fluorescence signal of chl-a of live cells during adverse growth conditions [67]. As the fluorescence signal depends on the properties of the specimens being cultivated and also on the culture conditions, it must be calibrated separately for different cultures [63]. Independently of the device used, IVF revealed the same patterns during culture development. Besides simple spectrofluorometers, an alternative is to use the minimum dark fluorescence F_0_ of PAM-fluorescence devices for IVF as long as the instrument settings are not changed during the measurement series. This is a promising approach because inexpensive devices are now available on the market.

### 3.7. Comparison of Biomass Parameters

**Figure 9:**
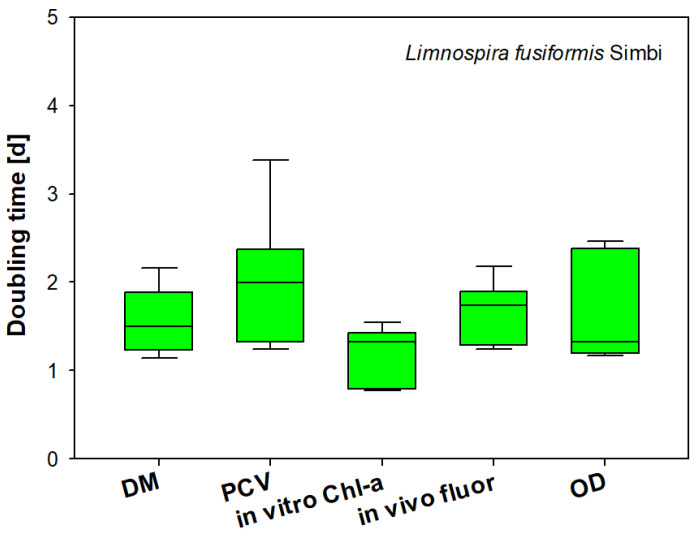
Comparison of biomass parameters during the exponential phase of batch cultures of *Limnospira fusiformis* (median box plots, n = 27).

In a growth experiment with the commercially exploited cyanoprokaryote *Limnospira fusiformis,* we compared direct and indirect parameters of algal biomass (Figure 9). As these parameters are based on different units hindering direct comparisons, we calculated doubling times from the respective parameter (Figure 9). We found similar results, with only in vitro chl-a showing significantly lower doubling times, which could be traced back to variations in cellular pigment content during culture development (Kruskal–Wallis test, n = 27, H = 30,93). Besides PCV, OD also has a large variance, which probably reflects improper readings from the hematocrit tubes (PCV) and varying optical properties due to changes in cell morphology (OD).

### 3.8. Vitality of Microalgae via PAM-Fluorescence

Optical measurements are strongly influenced by cell properties, and methods based on fluorescence are prone to IFE. This raises the fundamental question whether Fv/Fm is a robust parameter along different cell densities. This question is crucial because some devices on the market were developed for higher plants, which contain large amounts of pigments compared to suspensions. This implies that quite concentrated suspensions are necessary for stable readings. Moreover, new technologies and cheap components enabled the development of inexpensive battery-operated, handheld devices that also pave the way for use by non-academic institutions. Although Fv/Fm is not suitable for calculating growth rates and modelling primary productivity [67], it is a valuable biomarker to gain insight into the health status of oxygenic photoautotrophs during active growth [10,12,14,68,69] and storage [70].

**Figure 10:**
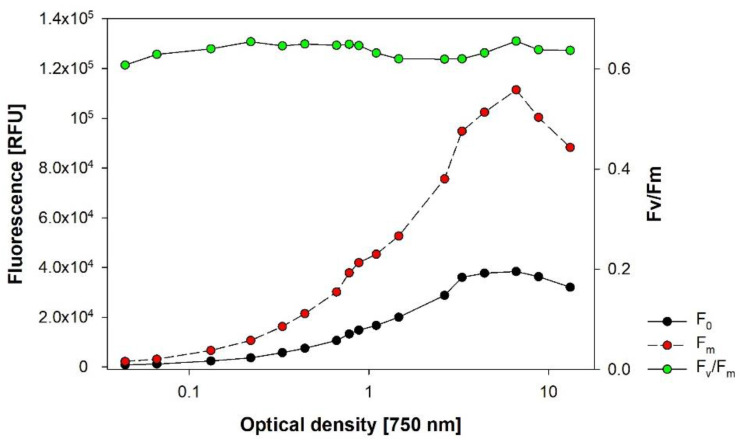
Dilution experiment with *Chlorella vulgaris* cultivated in BG11 full medium. The highest OD of about 13 corresponds to DM of 0.84 gL^−1^. Readings were taken with the AquaPen AP100 device.

In a dilution experiment performed with *Chlorella vulgaris*, we recorded the signals for F_0_ and F_m_ over a broad range of OD. As expected, we found the characteristic hump-shaped curve caused by the IFE, which was already observed in the IVF measurements (Figure 10). Fv/Fm, however, resulted in comparable values across almost the full range of OD (Figure 10), which is explained by mutual effects: both signals are affected in the same way, canceling the single IFE.

**Figure 11:**
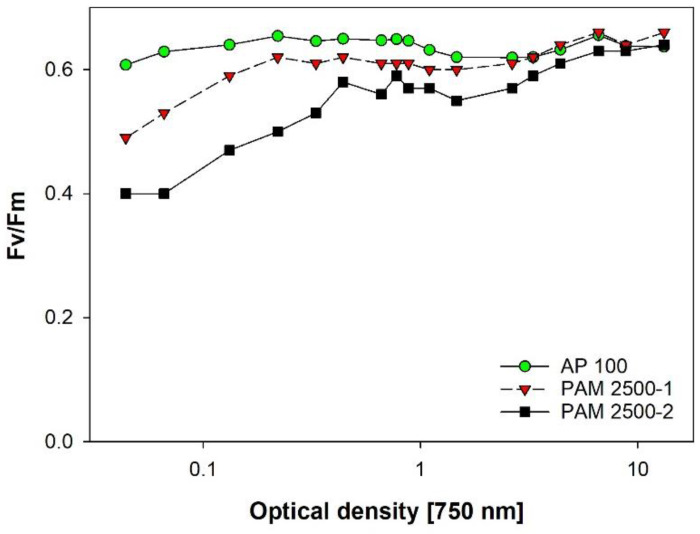
Comparison of Fv/Fm over a wide range of OD obtained with three PAM-fluorescence devices (highest OD of 13 corresponds to DM of 0.84 gL^−1^). Dilution experiment with *Chlorella vulgaris* cultivated in BG11 full medium.

The comparison of three devices revealed an interesting pattern: as expected, PAM2500-2 equipped with a fiber-optic cable and low sensitivity showed reliable results only at OD > 1 (Figure 11). The generally low values compared to the other instruments are caused by the different optical geometry. PAM2500-1 was specifically designed for diluted algal and chloroplast suspensions. The cuvette holder allows perpendicular excitation and emission light paths, and the high-sensitivity detector enables stable signals even at low cell densities, which also minimizes the IFE and scattering effects. The AquaPen AP100 showed a surprisingly even signal over a wide range of OD (Figure 11). We assume that the specific geometry of the optical path—with the excitation light and the fluorescence detector directly attached to the cuvette holder—is responsible for this excellent performance.

## 4. Conclusions

**Figure 12:**
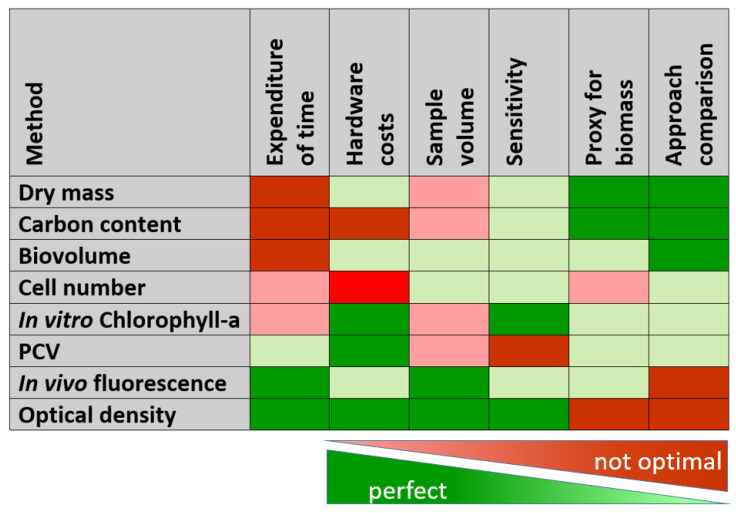
Summary of commonly used methods for estimating microalgal biomass (PCV = packed cell volume, in vitro Chlorophyll-a = spectrophotometric chl-measurement after solvent extraction). Colors represent the suitability for the respective property.

Methods with high accuracy and sensitivity are time-consuming, expensive and require sophisticated hardware and qualified personnel. Immediately available results often come at the expense of reliable conclusions (Figure 12). To overcome the limitations of easy-to-measure parameters, methods can be combined to enhance the informative value and reliability. For instance, daily measurements of OD and IVF for monitoring cultures can be calibrated against gravimetric methods, which are applied in weekly intervals.

Absolute parameters for algal biomass estimation are too laborious and time-consuming to test for daily monitoring. OD is a robust, fast and easy-to-measure parameter, but caution must be exercised because it is not specific for photoautotrophs. OD also demands specific requirements at higher cell density. IVF is an ideal proxy for fast and reliable data generation, but it bears the problem of IFE. The challenges of OD and IVF can be countered with appropriate dilution. A rule of thumb for 10 mm light path is diluting to an OD between 0.2 and 0.8.

Although physiological measurements based on fluorescence techniques face the same problem of IFE, Fv/Fm turned out as a very stable parameter across different concentrations of algae cultures because F_0_ and F_m_ are impaired in the same way. For routine analyses, inexpensive instruments that meet the demands of monitoring purposes are on the market. Moreover, F_0_ can be taken as the IVF signal, as long as the instrument setting is not changed during culture development.

## Figures and Tables

**Table 1:** Comparison of parameters for algal biomass estimation.

Causally Linked to Biomass—Absolute Units	Dry Mass
**Derived—absolute units**	Biovolume Packed cell volume Cell number Carbon content In vitro Chl-a
**Derived—relative units**	Optical density In vivo fluorescence

## Data Availability

The data presented in this study are available on request from the corresponding author.
